# Follower ants in a tandem pair are not always naïve

**DOI:** 10.1038/srep10747

**Published:** 2015-05-29

**Authors:** Patrick Schultheiss, Chloé A Raderschall, Ajay Narendra

**Affiliations:** 1 Research School of Biology, The Australian National University, Canberra, ACT 0200, Australia; 2 Department of Biological Sciences, Macquarie University, Sydney, NSW 2109, Australia

## Abstract

In addition to foraging individually several species of ants guide nestmates to a goal by tandem running. We found that the Australian ant, *Camponotus consobrinus*, forages both individually and by tandem running to head to the same goal, nest-specific native Australian trees on which they forage. While paths of solitary foragers and initial paths of tandem followers showed no differences in heading directions or straightness, tandem followers moved at about half the speed of solitary runs. When leaders were experimentally removed, follower ants initially engaged in a systematic search around the point of interruption, following which they either (a) headed directly towards and successfully reached the foraging trees, or (b) continued searching or (c) returned to the nest. The high incidence of followers that successfully navigated towards the foraging trees on their own provides strong evidence that many tandem followers are in fact experienced foragers. Detailed analysis of the searching behaviour revealed that even seemingly lost followers displayed a directional bias towards the foraging trees in their search path. Our results show that in a foraging context follower ants in a tandem pair are not always naïve.

Insects use a variety of cues to navigate to their places of interest. In ants, navigation behaviour has been well studied in solitary foraging species (e.g., *Cataglyphis*, *Formica, Melophorus*, *Myrmecia*). From these we know that individual animals are competent navigators deriving directional information from celestial and terrestrial cues and distance information from self-generated motion cues^1^,^2^. In several species of ants where solitary foraging is not the only navigation strategy, scout ants upon finding a new resource (nest/food), return to the nest to recruit nestmates. Using trail pheromones is the most sophisticated recruiting behaviour, but in some species nestmates guide each other to a goal either by carrying or by tandem running. Carrying behaviour typically occurs during nest emigrations where nestmates are either grasped and dragged (e.g., Myrmeciinae^3^) or carried either above (e.g., Myrmicinae, some Ponerinae) or below the transporter (e.g., Formicinae, some Ponerinae)^4^,^5^. Tandem running is a recruitment strategy that ants use to guide nestmates to new nest locations and to new food sources^6^,^7^ and is prevalent across the ant phylogeny (*Camponotus* spp.^8^,^9^, *Cardiocondyla* spp.^7^, *Temnothorax* spp.^10^,^11^,^12^, *Diacamma rugosum*^13^,^14^, *Harpagoxenus sublaevis*^6^, *Pachycondyla obscuricornis*^15^, *Pachycondyla tesserinoda*^16^ and *Polyrhachis proxima*^17^).

During tandem running, typically a pair of workers leave the nest with the leader remaining perfectly still until she receives a tap on the gaster from the follower’s antennae. The leader runs for a short distance (a few centimetres), pauses and upon receiving another tap moves again^8^. The tandem pair are bound to each other by this regular tactile cue and also by a surface pheromone discharged by the leader, but not necessarily by vision^18^. Since only one nestmate is recruited at a time, tandem running has been considered to be a primitive form of recruitment^7^,^8^. Tandem running has most often been studied in the context of nest relocation, where a scout ant having discovered a new nest site, returns home to recruit nestmates to this new location^14^,^19^. Nest relocation has mostly been studied in artificial nests in the laboratory^10^,^20^,^21^,^22^ and only recently in natural conditions^14^. From nest relocation studies carried out in the laboratory we know that followers of a tandem pair upon successfully reaching a goal, subsequently become leaders in another tandem pair^11^, suggesting that followers acquire navigational information during a tandem run. This can be tested by experimentally removing the leader in a tandem pair. From two lab-based nest relocation studies we know that the follower ants of an interrupted tandem pair (*Temnothorax albipennis*) either initially exhibit a search (sub-diffusive or random) and return to their old nest^21^ or followers continue searching and extrapolate their search to head towards the new nest, despite not having been there previously^22^.

Here, we investigate the navigational knowledge of followers and the information they have learnt during a tandem run while foraging in natural conditions. The Australian Banded Sugar ant, *Camponotus consobrinus* (Erichson) (Fig. 1), travels either individually or in tandem pairs to nest-specific native trees (*Casuarina* or *Eucalyptus)* on which they forage^23^. This provides us with an opportunity to identify in the ant’s natural habitat the navigational knowledge of followers in a tandem pair in a foraging context. We followed tandem pairs from the nest as they head to nest-specific trees for foraging using a Differential GPS^24^,^25^. We interrupted the tandem pairs about half-way to the goal by removing the leader ensuring the follower was not disturbed. We continued to track the followers to determine their navigational knowledge, specifically, their ability to find the location of the nest or the foraging tree. If the followers are truly naïve, we expect them to search extensively at the location where they lost contact with the leader and if the followers are experienced we expect them to head directly towards their nest-specific foraging trees.

## Results

In the southern hemisphere summer, *C. consobrinus* foragers begin their outbound foraging close to sunset. While many foragers leave the nest individually, about 2-35% head out foraging as tandem pairs. We filmed outbound activity before ants left the nest until no activity occurred over a 30-minute period. We found that outbound foraging activity of solitary ants occurred between 62.5 ± 28.6 minutes (mean ± s.d., n = 8) before sunset to 46.25 ± 7.44 minutes after sunset. On the other hand, outbound foraging activity of tandem pairs occurred between 50.0 ± 22.03 minutes before sunset to 20.0 ± 9.25 minutes after sunset. Thus outbound foraging activity of tandem pairs occurs mostly in the brighter part of the evening with their numbers rapidly decreasing after sunset (Fig. 2). Both solitary foragers and tandem pairs headed towards three trees located nearly 20 m from the nest, on which they foraged at night. As these trees were close together, and the foraging runs were well directed, the natural outbound traffic of the nest was restricted to a single 45° sector (Fig. 3a).

In 26 tandem pairs, we tracked the path of the follower ants to the half-way point between the nest and tree (blue circles in Fig. 3a) where we interrupted the tandem runs by removing the leader and then continued to track the follower. Two of these interruptions occurred naturally within 5 m of the nest entrance (Fig. 3a). After interruption, a high proportion of followers (40%; 11 of 26) after a very short search (260 ± 152 sec; mean ± s.d.; range: 114 – 593 sec; n = 11) headed towards and reached the foraging trees and were classified as ‘successful followers’ (Fig. 3b). A similar number of followers (40%; 11 of 26) searched extensively (574 ± 399 sec; mean ± s.d.; range: 253 – 1193 sec; n = 11) but reached neither the trees nor the nest within the time limit of recording (1200 sec), and were classified as ‘lost followers’ (Fig. 3c). A small proportion of followers (only 2 out of 26) returned to the nest after a brief search (226 seconds; 462 seconds) and were classified as ‘nest returnees’ (Fig. 3d). Two other followers displayed unusual behaviour: following interruption at the half-way point one follower encountered another tandem pair, displaced the follower and followed this leader ant to head to the tree in a tandem run (red path in Fig. 3e); another follower was led by its leader in a very short loop around the nest entrance and back into the nest (black path in Fig. 3e), a behaviour reminiscent of the learning walks in solitary foraging ants^26^. Since only two ants exhibited such unusual behaviour, we did not analyse these paths further.

We measured different characteristics (heading direction, sinuosity and walking speed) of the follower’s path by dividing each path into three segments: (a) segment one – the natural part of the tandem run from the nest entrance to the interruption point; (b) segment two – the search phase from the interruption point to the end of search, defined as the location where the ant initiated movement in either a homeward or treeward direction (i.e., in either direction of segment one) and kept this heading for a minimum of two metres; (c) segment three: remainder of the path. In cases where an ant continued searching for the entire recording duration (20 minutes after interruption) segment 3 was absent (see methods for more details).

In segment one, the heading directions of followers in all three groups, i.e., successful, lost and nest returnees, were well directed towards their foraging trees (Fig. 4, first column). In segment two, the heading direction of followers in all three groups (successful, lost and nest returnees) had a wide spread, which is characteristic of the search behaviour. Some ants exhibited a tendency to head either towards the foraging trees (0°) or towards the nest (−180° or +180°). In segment three the heading direction of followers (a) of the successful group was directed towards the trees, (b) of the lost group was directed either towards the nest or tree, (c) of the nest returnee group was directed towards the nest. Four followers of the lost group continued searching for the entire recording duration and hence lack data from segment three. One ant of the nest returnee group returned to the nest through a continued search and also lacks data for segment three.

As very few ants returned to the nest, only the paths of the successful and lost groups were analysed further. Path straightness of successful and lost followers did not differ in segment one (*t*-test; *p* = 0.75) and in segment two (*t*-test; *p* = 0.1) (Fig. 5a). However, in segment three, paths of the successful group were straighter than the paths of the lost group (*t*-test; *p* < 0.05). By comparing these to solitary foraging *C. consobrinus* ants, it is evident that the path straightness of solitary ants is similar only to segment one of both the successful (*t*-test; *p* = 0.14) and the lost group (*t*-test; *p* = 0.08). Segments 2 and 3 of both successful and lost followers were considerably less straight than solitary runs (*t*-tests; all *p* < 0.001).

Walking speed of successful and lost followers did not differ in segment one (*t*-test; *p* = 0.74; successful: 2.67 ± 0.46 cm/s; lost: 2.58 ± 0.71 cm/s; Fig. 5b) and in segment three (*t*-test; *p* = 0.95; successful: 3.72 ± 1.1 cm/s; lost: 3.78 ± 2.72 cm/s). In segment two, which captures the search behaviour of ants, walking speed of successful followers was greater than the walking speed of lost followers (*t*-test; *p* < 0.01; successful: 5.25 ± 1.14 cm/s; lost: 3.88 ± 0.86 cm/s). Solitary ants in comparison walked faster (5.61 ± 1.5 cm/s) than both groups of follower ants in segment one (*t*-tests; both *p* < 0.001), faster than lost but not successful followers in segment two (*t*-tests; solitary vs. successful: *p* = 0.49; solitary vs. lost: *p* < 0.01), and faster than successful but not lost followers in segment 3 (*t*-tests; solitary vs. successful: *p* < 0.01; solitary vs. lost: *p* = 0.11).

We analysed the search pattern of the followers in more detail. In all cases, immediately after the removal of the leader (both experimental and natural breaks) follower ants engaged in a searching behaviour. Search duration of the lost and successful followers differed significantly (*t*-test: *p* < 0.05). As searches of lost followers lasted longer than those of successful followers, we restricted our analyses to the first two minutes of search (Fig. 6a, red paths). Over time, searching follower ants ventured further away from the point of interruption, but also returned to this point repeatedly (Fig. 6b). We measured this search perseverance by determining the number of times an ant returned to the point of interruption within the first two minutes. We found no difference in the search perseverance of followers between the successful and the lost group (*t*-test; *p* = 0.24), although successful ants returned to the point of interruption more often than lost ants (mean ± s.d. successful followers: 2.8 ± 2.04, lost followers: 1.67 ± 2). In most follower ants, the centre of search was not located at the point of interruption. Instead, there was a drift in the continued direction of the initial tandem run, away from the nest. Figure 6c shows that this shift along the axis of the initial tandem run gradually increased over time. It appears to reach a plateau in the lost follower group after about a minute of searching, but steadily increases in a linear fashion in the successful follower group.

## Discussion

The crepuscular ant *C. consobrinus* forages both individually and as tandem pairs to head to nest-specific native Australian trees on which they forage the entire night. Tandem runs occurred frequently throughout the period of foraging activity with maximum pairs leaving the nest just before sunset. We showed that in nearly half of the interrupted tandem pairs, follower ants directly head to their goal (segment three in Fig. 4) providing strong evidence that followers in a tandem pair are not naïve to the food source. If these successful animals were naïve they would be able to reach their goal only through a search strategy^22^, which was not the case here.

So why do some experienced ants that have the navigational knowledge of the foraging tree act as a follower in a tandem run? This is surprising since tandem running appears to be costly, as walking speed of followers during tandem running is reduced by nearly 50% compared to solitary foraging ants (Fig. 5b). One likely explanation is that animals that have previously visited the tree to attend one food source are guided by tandem running to a different foraging location on the same tree. This is perhaps because the tree itself is a complex foraging structure with the locations of food resources often changing, which requires acquisition of new navigational knowledge. Thus, even experienced foragers with a detailed knowledge of the terrestrial environment will have to learn spatial information of the new food location on the tree. This hypothesis can be tested by marking leaders of all tandem runs and then blocking access to the food source and providing animals with a new food source. Preliminary evidence^23^ from such an experiment suggests that former leaders do become followers in the tandem runs leading to the new food source. The navigational challenge of followers in a tandem run appears to be slightly different in the context of foraging and nest relocation. During nest relocation, followers in a tandem pair are naïve to the new nest location and when their tandem runs are interrupted and leaders are removed, the majority of the followers return to the old nest. The few followers that find the new nest arrive there through a directionally biased search^21^,^27^ but never reach it in a straight path, which is in contrast to the successful *C. consobrinus* followers (segment 3 in Figs. 4,5).

Search strategy of followers in a tandem run: When tandem runs were experimentally interrupted, followers immediately engaged in a searching behaviour, returning regularly close to the point of interruption, most likely in an attempt to re-establish contact with the leader (Figs. 4,5). About half of the follower ants could not find their way to either the trees or to the nest (Fig. 3c) and another half of the followers found their way to the foraging trees (Figs. 4,5). The ‘lost’ followers engaged in prolonged searching behaviour (about 10 min duration on average), while the search phase of successful followers was much shorter (about 4.5 min on average). During the search the walking speed was significantly lower in ‘lost’ followers (Fig. 5) and the pattern of directional bias in the search also differed between the two groups (Fig. 6c). In the successful followers, the entire search path gradually drifted in the direction of the previous tandem run in a linear fashion, while this drift plateaued after about a minute in the lost followers. Taken together, these differences indicate that lost followers search for their leaders with much more persistence than successful followers and are perhaps naïve in contrast to the other followers.

The presence of the directional bias itself is interesting, as all followers, including those of the lost group, seem to have acquired some sense of directionality. Learning the directional heading from the initial uninterrupted phase of the tandem run, combined with an ability to extrapolate in this direction after separation could explain this behaviour. Under natural conditions such a behaviour makes perfect sense as undisturbed tandem runs are frequently separated for a brief period when the gap between the leader and follower ants becomes too large and they lose direct physical contact. For the follower, the chances of finding the leader ant will therefore be greater in a forward direction than in a backward direction. Such an ability of follower ants to extrapolate the direction of the initial tandem run, leading to a directional bias in their search, has previously been described in *T. albipennis*^21^,^27^.

Role of vision in tandem running: Evidence that followers in a tandem pair rely on visual cues for navigation comes from the fact that after interruption some followers, (a) head directly to their foraging trees, (b) return to the nest, or (c) engage in a search, during which they regularly return to the point of interruption. To achieve these navigational tasks animals must use a visual compass that could comprise familiar terrestrial landmarks or the pattern of polarised skylight. *Camponotus consobrinus* have over 700 facets in each eye with an interommatidial angle of 5.2°, sufficient to detect visual information (A. Narendra pers. obs). This is comparable to the intertidal ant *Polyrhachis sokolova* (600 facets; 5.9° interommatidial angle) that also relies on visual information for navigation^28^,^29^. *Camponotus consobrinus* have specialised photoreceptors in the dorsal rim area (DRA) of the eye to detect changes in the pattern of polarised skylight (A. Narendra pers. obs). In the DRA, the rhabdoms are rectangular in shape and the microvilli of the retinular cells in each ommatidium are oriented 90° to each other, which is an anatomical specialisation required for detecting polarised skylight^30^. The role of vision in tandem running has been addressed in *T. albipennis* by blocking the eyes of either the followers or the leaders or both^18^. Under the laboratory conditions where these experiments were carried out, vision did not play a significant role, with tandem runs being established even by a blind leader. However, a vision-impaired follower slowed down the walking speed of a tandem pair compared to a fully sighted follower. These ants perhaps rely on other sensory cues (e.g., tactile and chemical) during their tandem run, especially since they engage in a task of nest relocation that is strikingly different from the task of *C. consobrinus* that involves repeatedly visiting a specific foraging location. Further evidence that *C. consobrinus* rely on visual cues during navigation comes from their activity schedule. Tandem runs occurred mostly in the brighter part of the evening with none being formed 20.0 ± 9.25 minutes after sunset, perhaps because light intensity drops dramatically during evening twilight, decreasing the salience of visual navigational information. It remains to be seen whether it is the leader’s or the follower’s inability to access sufficient information that restricts tandem runs to brighter light conditions. Lastly, one of the tandem pairs we recorded exhibited a behaviour similar to that displayed by a forager leaving the nest for the first time. The tandem run began at the nest, travelled less than 1m from the nest in a loop and returned back to the nest (Fig. 3e). Ants leaving the nest for the first time are known to carry out such a learning walk during which individuals most likely acquire sufficient landmark information that would assist in homing^24^,^26^. Walking in tandem during a learning walk may allow the follower to acquire landmark information around the nest, while a tactile contact with the leader ensures she returns to the nest at the end of the learning walk. We recorded only one tandem learning walk, perhaps because this is a rare phenomenon or due to the absence of naïve animals leaving the nest in our experiments.

## Materials and Methods

### Study species and location

**Figure 1:**
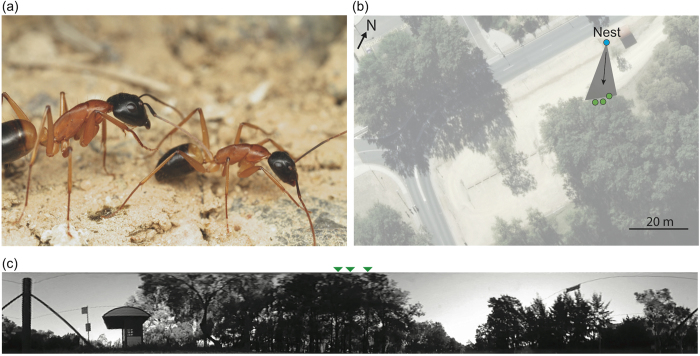
Tandem running in sugar ants. (**a**) Pair of workers of *Camponotus consobrinus* engaged in tandem running with the leader on the right. (**b**) Study location showing nest site and three foraging trees (green circles). Foraging corridor is shown in grey shaded area. Arrow indicates typical foraging direction. (**c**) Panoramic view available to the ants from the nest, showing the three tree locations (green arrows). Photo credit: Ajay Narendra.

**Figure 2:**
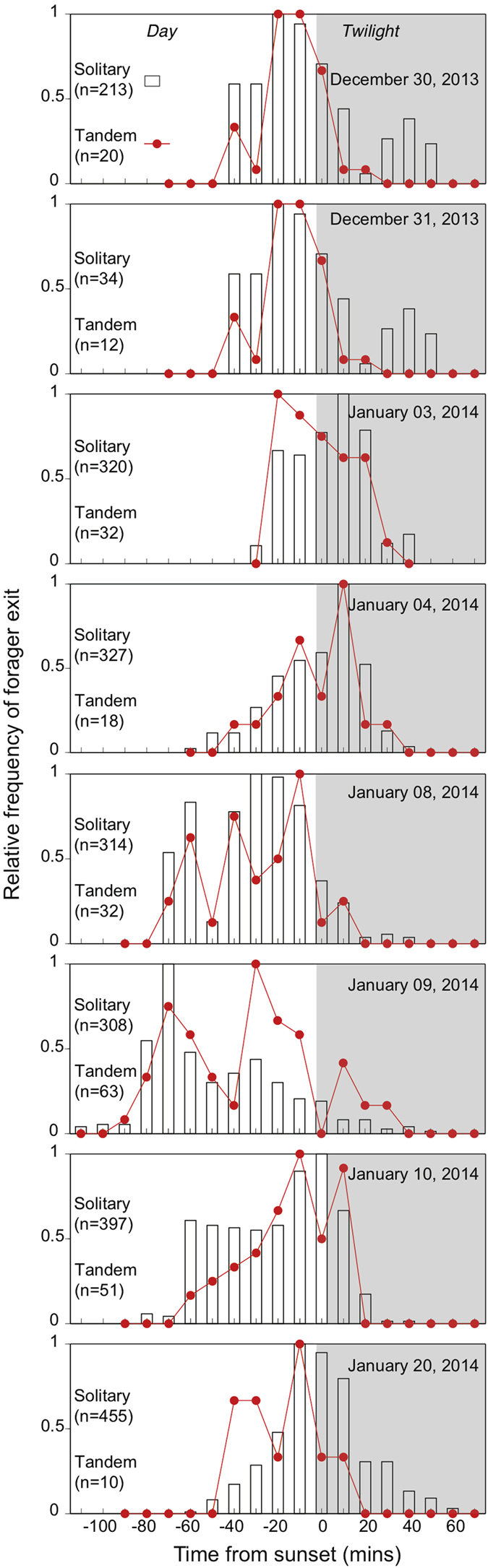
Outbound activity patterns of *Camponotus consobrinus* foragers from a single nest on eight days. Recording started two hours before sunset, and continued until about one hour after sunset. Solitary foragers (bars) and tandem runs (red dots and line) are shown normalised to their respective maxima. The grey shade indicates the period after sunset.

**Figure 3:**
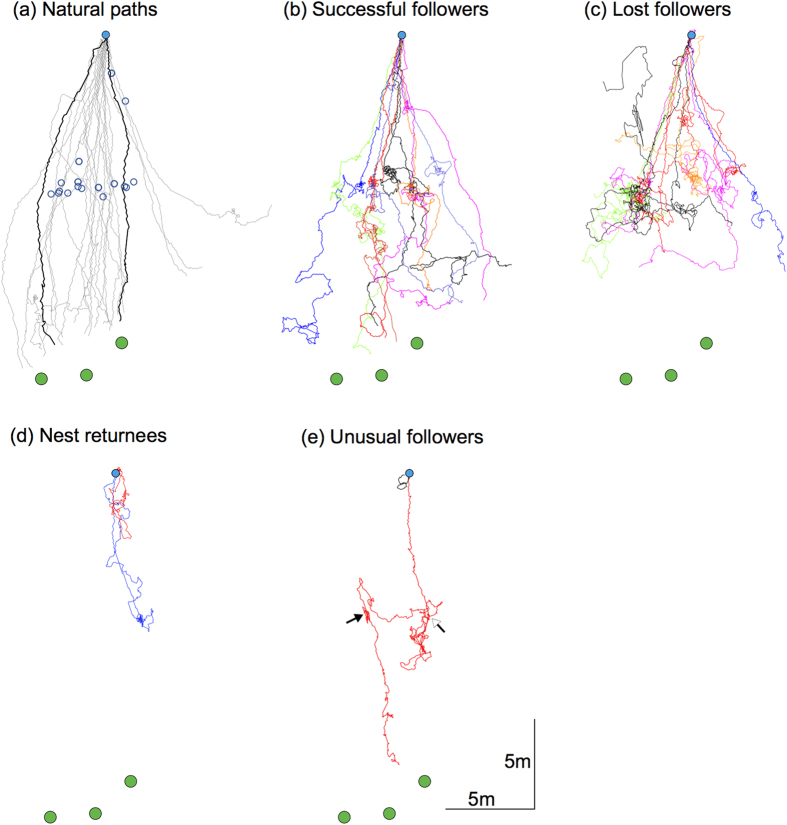
Paths of *Camponotus consobrinus* foragers. (**a**) Natural, undisturbed paths of solitary foragers (grey lines) and two tandem runs (black lines). Nest (filled blue circle), three foraging trees (green circles), interruption points where leaders where removed in later conditions (open blue circles). After leader removal, paths of followers (**b**) that successfully reached the foraging trees, (**c**) that did not reach either the trees or the nest, (**d**) returned to the nest. (**e**) In red, path of a follower after interruption (open arrow), that displaces a follower of another tandem pair (closed arrow) and heads to the tree; in black, a tandem pair leaves the nest and returns to the nest entrance. Colours represent different ants.

**Figure 4:**
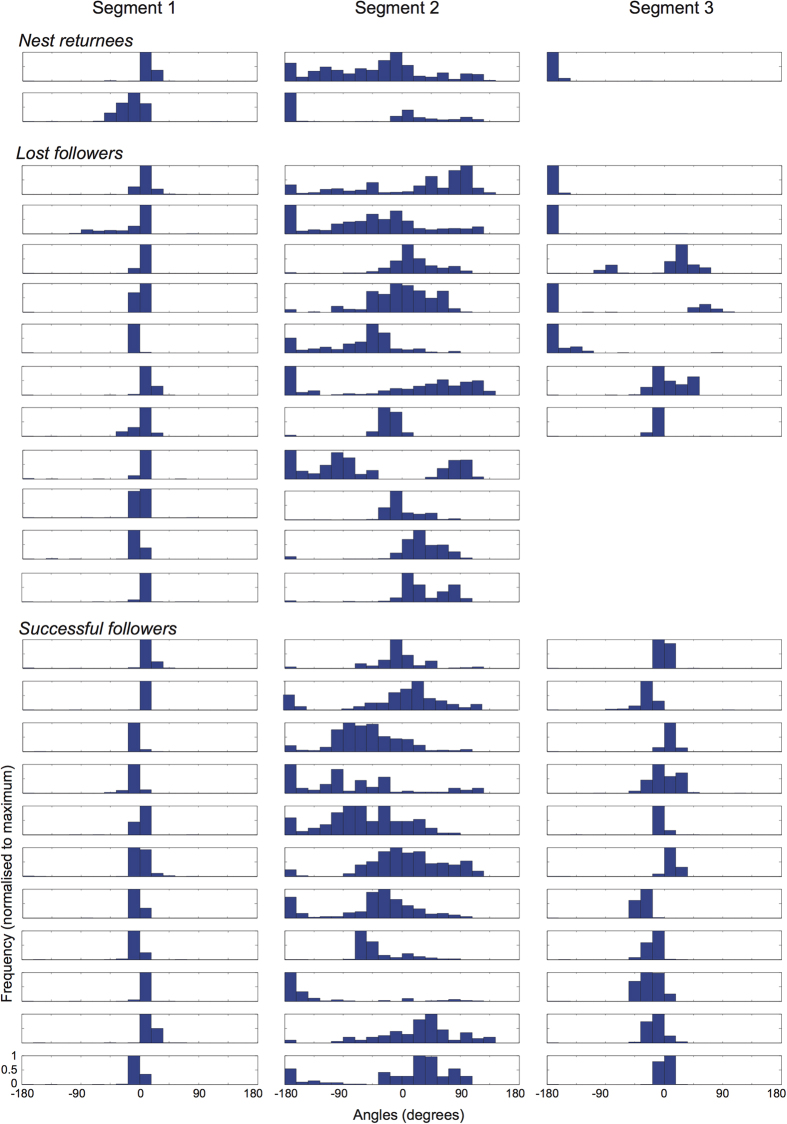
Histograms of heading directions of follower ants during interrupted tandem runs, separated into three segments. Segment 1: tandem run until interruption; segment 2: the search phase after interruption; segment 3: the final part after the end of search, if present. Each subplot has been normalised to the respective maximum. 0° = bearing from nest to foraging tree.

**Figure 5:**
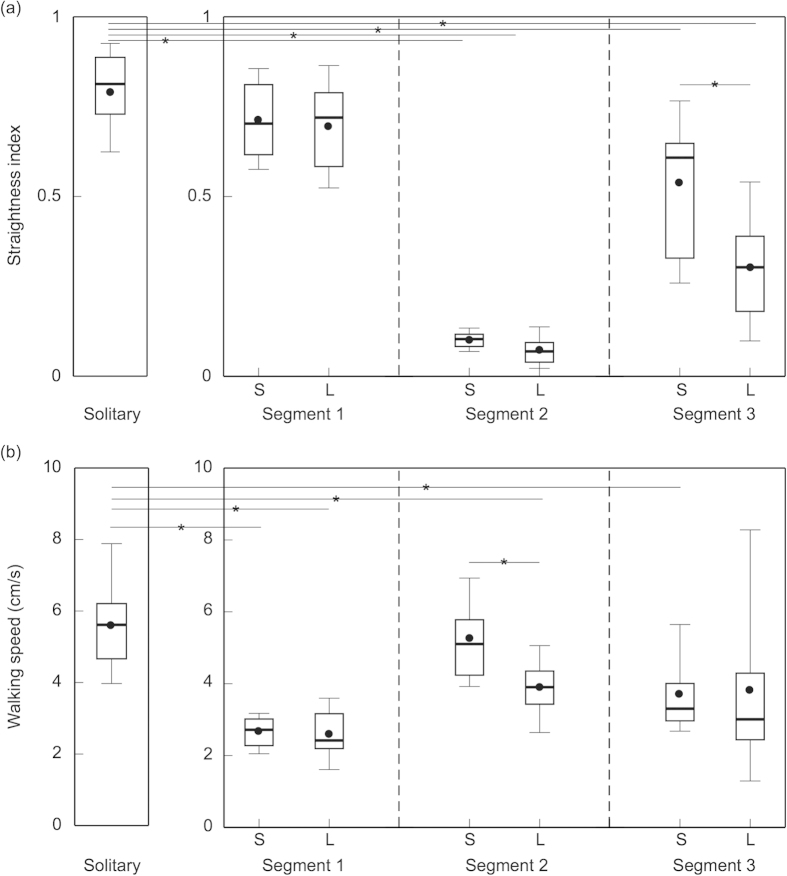
Path measures of solitary foragers and followers from interrupted runs. (**a**) Path straightness and (**b**) walking speed of solitary foragers (left) and followers from interrupted tandem runs (right), separated into three path segments. Boxes show median, upper and lower quartile, whiskers extend to upper and lower deciles. Average values are shown as black dots. The small star denotes significant differences between groups. S: successful followers, L: lost followers.

**Figure 6:**
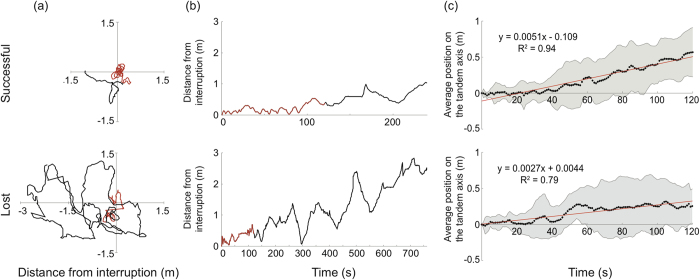
Searching behaviour of tandem followers after interruption of the tandem run. (**a**) Example search paths, with the first two minutes of search shown in red. (**b**) Distance from interruption point over time, derived for the example path shown in (**a**). The first two minutes of search are shown in red. (**c**) Average position along the axis of the preceding tandem run, over the first two minutes of search for all tandem followers. Positive values are in the direction of the tandem run, negative values are in the direction back to the nest. Grey shading shows the standard deviation, red line shows the best linear fit.

We studied the Formicine ant, *Camponotus consobrinus* (Erichson), commonly known as the Australian Banded Sugar ant (Fig. 1). This study was carried out at a single nest located in an open car park at The Australian National University, Canberra (35°16’44.72”S, 149° 7’5.02"E). These are ground nesting ants with nest entrances characterised by a small mound, or a simple circular hole in the ground or underneath small stones. The body size of the foraging ants ranged between 5 - 11 mm, with foragers typically returning home with bird droppings and honeydew collected from aphids and mealy bugs. During summer, these ants typically start foraging an hour before sunset with most ants returning to the nest within two hours after sunrise.

### Activity monitoring

On the days when we recorded the ant paths, we also set up a digital video camera (SONY HDR CX700VE) and filmed the nest from above to record the time at which individuals and tandem pairs exited the nest. We digitised the videos using Final Cut Pro X (Apple Inc.) and recorded the exit times in 10-min bins.

### Experimental setup

We tracked outbound paths of solitary foragers and tandem pairs from a single nest as they headed towards their foraging trees. For 26 tandem pairs, at about the half-way mark between the nest and the tree, we removed the leaders, taking great care to ensure the follower was undisturbed. The path of the follower was tracked until animals reached the nest or the tree or for at least 20 minutes after interruption. We removed conspecifics travelling along this route when they came within 15 cm of the follower to avoid nestmate interaction. Dense leaf litter at the base of the tree prevented us from collecting data about 1m from the tree, but we confirmed the ants climbed the trees by eye. Thus, ants were assigned to one of three categories: successful followers when they continued to and reached the tree; lost followers when they reached neither the tree nor the nest; and nest returnees when they returned to the nest. Ants were tracked using a Differential GPS, from which we acquired positional data every 1s^24^,^29^. We monitored the errors and ensured the accuracy was 20 cm or better (in most cases it was 3-10 cm).

For further analysis, the path of each follower was separated into three segments as follows: segment 1 encompassed the natural part of the tandem run; it started as soon as the tandem pair was formed very close to the nest entrance, and ended at the point where the leader was removed (the interruption point). Segment 2 encompassed the search phase, where the follower was attempting to locate the leader; it began at the interruption point. Due to the variability of the followers’ behaviour, the endpoint of the searching phase was sometimes hard to distinguish by visual means alone, and required a clear quantitative measure. Reasoning that the searching follower ants had three different choices (go home, go to the tree, continue searching), we considered only displacements along the axis of the preceding segment 1 (the tandem run direction); the endpoint of segment 2 was then defined as the point at which the ant initiated a change in heading in either a homeward or a treeward direction (as defined by segment 1) and kept this heading for a minimum of two metres. Segment 3 then encompassed the remainder of the path from this point onwards, beginning with the two or more metres of well-oriented path. If an ant did not reach the criteria for the end of search, segment 2 extended until the end of the recorded path.

For each of the three groups of ants (nest returnees, successful, and lost) and for the three segments we measured (a) heading direction, (b) path straightness, and (c) walking speed. Heading direction was determined from positional data acquired at every one-second interval from the Differential GPS. Path straightness was measured by dividing the beeline distance between the start and end points by the length of the actual path taken by the ant. For all comparisons between groups we used the Student’s *t*-test. To investigate the navigational knowledge of the follower ants in detail, the searching phase of their paths (segment 2) was further scrutinised. For this we only considered ants that searched for ≥2 min. As a measure of search perseverance, we counted the number of times an ant returned to the point of interruption during the first 2 min. Each approach to within 10 cm or less of the interruption point was counted as a return, and two successive returns had to be separated by a ≥3 second interval. We identified where each ant focussed its search, by calculating the centre of search as the median of all *x* and the median of all *y* values. Finally, we investigated whether, as a group, follower ants showed any drift in their search over time towards either the trees or the nest location. For this, all paths of follower ants were rotated so that their segment 1 (the initial tandem run) pointed in the same direction. Along this axis, we then looked for a shift in either direction during the first 2 min of search.

## Additional Information

**How to cite this article**: Schultheiss, P. *et al.* Follower ants in a tandem pair are not always naïve. *Sci. Rep.* **5**, 10747; doi: 10.1038/srep10747 (2015).
