# Corrigendum: Follower ants in a tandem pair are not always naïve

**DOI:** 10.1038/srep29331

**Published:** 2016-07-08

**Authors:** Patrick Schultheiss, Chloé A Raderschall, Ajay Narendra

Scientific Reports 5: Article number: 10747; 10.1038/srep10747Published online: 05292016; Updated: 07082016

The authors neglected to cite a published thesis that investigated a related aspect of tandem running in the same study species. This additional reference is listed below as reference 1, and should appear in the text as below.

In the Introduction section,

“Tandem running is a recruitment strategy that ants use to guide nestmates to new nest locations and to new food sources^6,7^ and is prevalent across the ant phylogeny (*Camponotus* spp.^8,9^, *Cardiocondyla* spp.^7^, *Temnothorax* spp.^10–12^, *Diacamma rugosum*^13,14^, *Harpagoxenus sublaevis*^6^, *Pachycondyla obscuricornis*^15^, *Pachycondyla tesserinoda*^16^ and *Polyrhachis proxima*^17^).”

should read:

“Tandem running is a recruitment strategy that ants use to guide nestmates to new nest locations and to new food sources^6,7^ and is prevalent across the ant phylogeny (*Camponotus* spp.^1,8,9^ *Cardiocondyla* spp.^7^, *Temnothorax* spp.^10–12^, *Diacamma rugosum*^13,14^, *Harpagoxenus sublaevis*^6^, *Pachycondyla obscuricornis*^15^, *Pachycondyla tesserinoda*^16^ and *Polyrhachis proxima*^17^)”.

In the same section,

“Since only one nestmate is recruited at a time, tandem running has been considered to be a primitive form of recruitment^7,8^”.

should read:

“Since only one nestmate is recruited at a time, tandem running has been considered to be a primitive form of recruitment^1,7,8^”.
